# Sculpting with stiffness: rigidity as a regulator of morphogenesis

**DOI:** 10.1042/BST20220826

**Published:** 2023-04-28

**Authors:** Adam Shellard, Roberto Mayor

**Affiliations:** Department of Cell and Developmental Biology, University College London, Gower Street, London WC1E 6BT, U.K.

**Keywords:** development, mechanics, morphogenesis, rigidity, robustness, stiffness

## Abstract

From a physical perspective, morphogenesis of tissues results from interplay between their material properties and the mechanical forces exerted on them. The importance of mechanical forces in influencing cell behaviour is widely recognised, whereas the importance of tissue material properties *in vivo*, like stiffness, has only begun to receive attention in recent years. In this mini-review, we highlight key themes and concepts that have emerged related to how tissue stiffness, a fundamental material property, guides various morphogenetic processes in living organisms.

## Introduction

D'Arcy Thompson's *On Growth and Form* posited that biological form by morphogenesis is a direct reflection of physical and mathematical notions, thereby setting forward the principles of mechanobiology [1]. At its simplest, morphogenesis can be considered a direct consequence of the material properties of a tissue — such as stiffness, shear and viscosity — and the forces applied unto it. Both are normally heterogeneous and evolve over time. The advert of new technologies which can manipulate molecular governors of force has provided a route to understanding forces exerted by cells and tissues. By comparison, only in the last few years has there been considerable effort into understanding how material properties influence morphogenesis. There is thus a growing appreciation that tissue-scale mechanisms of behaviour are profoundly influenced by the physical nature of tissues [2].

In this mini-review, we will focus on how changes in stiffness can affect morphogenesis. Stiffness is one of the most studied material properties for biological systems and refers to the ability of a material to deform under load [3]. It is a passive mechanical property which resists deformation. Consider two materials of different stiffnesses. Under equal load (the force exerted on a surface or body), the soft material will deform more than the stiffer material. ‘Stiffness’ is often used interchangeably with the term ‘rigidity’ and we follow this practice in this article.

Biological tissues exhibit stiffnesses that can range over several orders of magnitude [4]. For example, neural tissue is 100-fold softer than smooth muscle, which itself is 10-fold softer than bone. Such heterogeneities in stiffness are also found at practically every scale, from organs to cells to individual fibres.

Both intracellular composition and extracellular environmental properties contribute to rigidity. Cellular properties that regulate stiffness include turgor pressure, cell density, the expression and organisation of cytoskeletal components, cortical tension, and the geometric configuration and orientation of the constituent cells within a tissue. Notably, myosin, which can generate cellular contractility, can also regulate the mechanical properties of the tissue — because myosin has the capacity to stiffen actin networks by remaining bound to actin, which prevents network fluidisation [5]. Also, these properties can enable a cell to be mechanically anisotropic [6]. Considering the extracellular environment, the composition and amount of ECM, the degree of cross-linking and degradation, and geometric constraints all influence tissue stiffness.

The ultimate stiffness of a tissue or organ is concordant with its function. Bone must be rigid to successfully act as a frame for the body, whereas soft tissues provide flexibility. However, stiffness is not only an output to serve the final function of a tissue. It is also intricately tied to the shaping of developing tissues and plays a role in guiding it, for example, via mechanotransduction signalling pathways (Table 1). Indeed, growth-promoting gene expression rarely correlates well with active cell expansion [7,8], suggesting that gene expression patterns alone are insufficient to predict the influence of genes on shape. Here, our aim is to discuss some emerging and generalisable functions of stiffness with respect to tissue morphogenesis.

**Table 1:** Mechanotransduction pathways

Mechanotransduction signalling pathway	Role in response to mechanical cues	Example reference
Hippo/YAP/TAZ	Proliferation, differentiation, organ size and tissue regeneration	[75]
MAPK/ERK	Survival, proliferation, differentiation	[76]
FAK-Src	Survival, proliferation, differentiation	[77]
PI3K/AKT	Growth, proliferation, survival	[78]
Rho/Rock	Actin cytoskeleton remodelling, adhesion, motility	[79]
Wnt/β-catenin	Prolfieration, differentiation, apoptosis	[80]
Twist	Epithelial-mesenchymal transition (EMT)	[50]

Cells can respond to mechanical stimuli via mechanotransduction signalling pathways, for example, stretch-activated ion channels can be opened in response to tension; forces can cause the cell membrane to stretch or deform, altering the activity of membrane associated-proteins, and forces can generate fluid flow through tissues.

## Constraining growth to shape form

**Figure 1.**
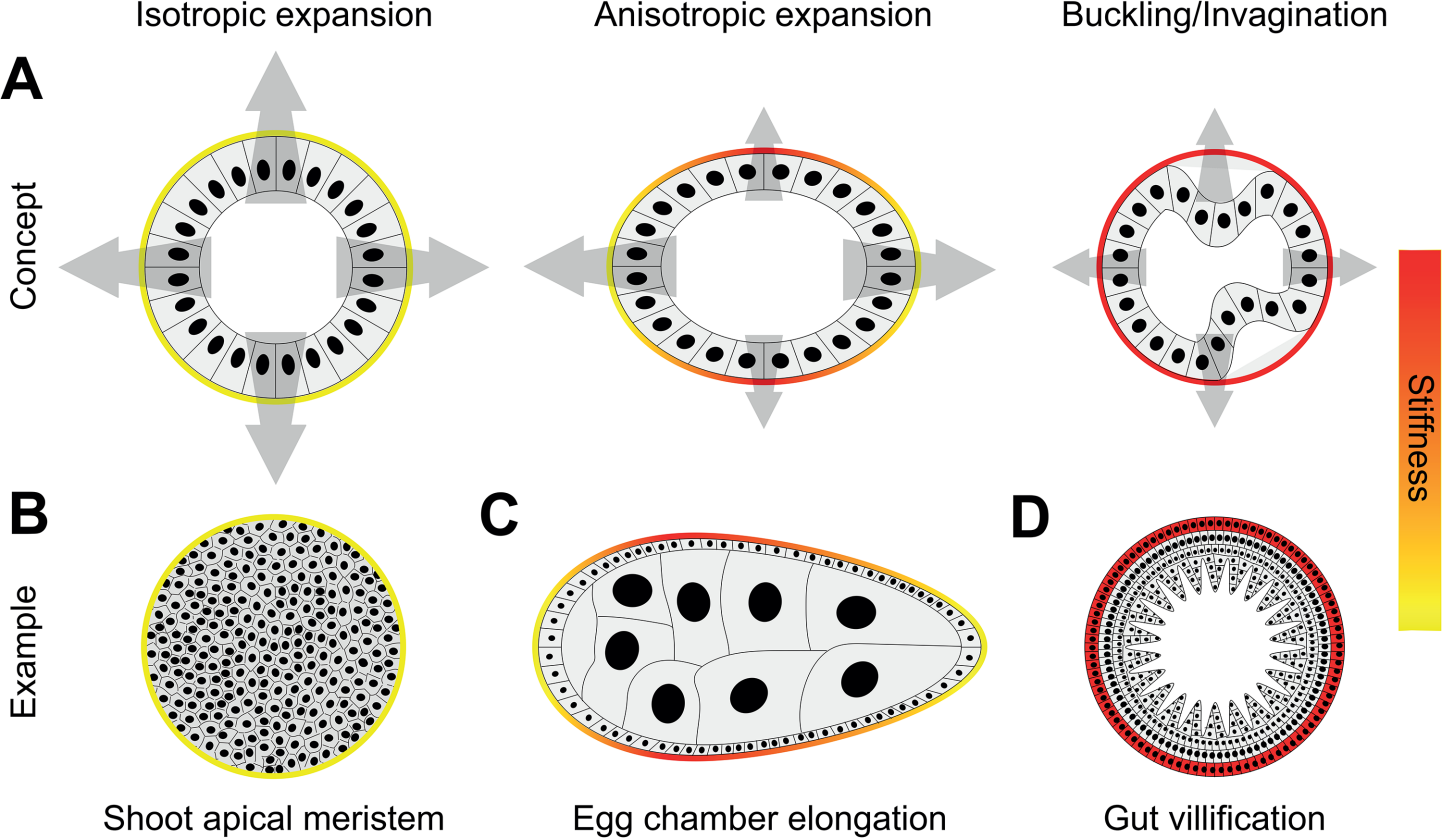
The stiffness of surroundings shapes tissues. (**A**) Cells growing and proliferating take up increasing amounts of space. If the surrounding environment is soft (yellow), a monolayer will expand (left). If the environment is mechanically patterned with soft and stiff (red) regions (middle), the monolayer will only expand into the soft regions, because they offer low resistance. This can make a tissue produce several different morphological forms, most typically elongating it. If the surroundings are rigid, the monolayer will buckle to relieve the tension, meaning it folds inwards (right). Examples of these are shown in **B**–**D**. (**B**) Roughly isotropic expansion of the shoot apical meristem by relatively uniform surrounding rigidity. (**C**) Elongation of the *Drosophila* egg chamber thanks to patterned ECM. (**D**) Villification of the gut is caused by a very stiff circular muscle layer forcing the mesenchyme and endoderm to buckle.

As cells grow and proliferate, they occupy more space. If they grow isotropically and in isolation, they form increasingly large spheres. However, *in vivo*, cells are not secluded. Rather, they have constraints from their surroundings. The physical characteristics of such constraints mould growing tissues. A tissue that grows in a soft environment can expand outwards because the boundary is deformable (Figure 1A, left). This can be evidently observed in culture when cell monolayers are grown inside soft capsules; cell proliferation expands the tissue without any folding [9]. In this case, there is little resistance provided by external components, so expansion is unimpeded.

The development of various organs is based on this principle. Expansion of the brain ventricle lumen during hindbrain morphogenesis is only possible through softening of the surrounding epithelium, and in stiff mutants this is impaired because the ventricle fails to enlarge [10]. Elongation of the vertebrate body axis is abrogated when constraints are uniformly soft, resulting in isotropic expansion instead of uniaxial lengthening [11]. In plants, the shoot apical meristem and leaf blade both have the potential to undergo isotropic growth and expansion (Figure 1B), thanks to hormones like auxin, which disorganise microtubules, thereby making filament orientation more random and consequently cell wall stiffness mechanically isotropic [12–15].

On the other hand, outward growth can be negated by a rigid outer shell, because the pressure of the growing tissue is insufficient to deform the surroundings. Tissues can thus be anisotropically shaped by constraining them within surroundings that have spatial heterogeneity in stiffness, since there will be differential resistance to the forces involved in growth. A tissue will expand into soft regions, and not into stiff regions (Figure 1A, middle).

Elongation of tissues and organs across varied systems and organisms is a process that arises because of this. The extension of the developing *Drosophila* egg chamber is driven by collective migration of follicular epithelial cells [16] that sense a surrounding ECM which is heterogeneously stiff [17]. The circumferentially aligned ECM has low rigidity at the poles of the egg chamber and high rigidity toward the centre [18]. Cells are re-oriented in a stiffness-dependent manner to elongate the organ at the poles [17]. Thus, a patterned differential resistance to luminal expansion promotes elongation, rather than isotropic expansion [18] (Figure 1C).

This type of physical constraint-driving elongation has been described as a biological corset that shapes a developing organ, and it is seen in many other situations [19,20]. During *C. elegans* axis elongation, the epidermal actin cytoskeleton is polarised, causing an anisotropy in stiffness that orients axis extension, promoting elongation over inflation [21]. Stiffness anisotropy may likewise help explain notochord elongation [22].

During culmination to form a fruiting body, *Dictyostelium* cells aggregate and the stalk grows upwards. This elongation is driven by a 5-fold increase in stalk cell volume which, because they are encased in a rigid stalk tube, expand unidirectionally, which contributes to the lifting force that raises the spore head [23,24].

In plants, patterns of morphogenesis are accounted for by mechanical anisotropy of the cell wall (caused by stiff cellulose microfibrils), which determine the direction for growth. Primordium growth in the shoot apical meristem is initiated by a local softening of the cell wall, leading to outgrowth of a well-defined bump [25]. For growth of the hypocotyl epidermis, stiff transverse walls restrict growth, whereas selective relaxation of longitudinal walls permit growth [26].

Recently, a completely different mechanism — yet still reliant on stiffness — has been described. During semi-circular canal morphogenesis, buds are surrounded by isotropic hyaluronate pressure, yet rise to elongated structures. Instead of patterned ECM rigidity shaping the tissue, cells are connected by a biased orientation of intercellular tethers — which provides rigidity — in the circumferential axis, and this anisotropic resistance to isotropic pressure directs the bud toward a tube. [27]

These wide-ranging examples reveal that mechanical heterogeneity in a tissue's surroundings, or patterned resistance (stiffness) to external pressure, instructs organ morphogenesis, and allows for a more holistic appreciation for how genetic and mechanical signals interplay to control morphogenesis, resolving conflicts between spatiotemporal patterns of gene expression and organ growth [28].

Next, one can consider a situation in which a tissue experiences stiff constraints. In this situation, if the tissue has some epithelial organisation, substantial growth will ultimately cause buckling, which refers to a sudden change in shape of a component due to load. It presents as a bending or local collapse of the structure due to pressure. The effect is seen when cell monolayers are grown inside elastic spherical shells; the monolayer relaxes excess proliferation by buckling (Figure 1A, right) [9].

Buckling by this mechanism is seen in various morphogenetic processes *in vivo*. The initially spherically shaped optic vesicle is constrained by ECM, which prevents it from expanding further outwards. The result is an inwards bending (i.e. invaginate) to create the optic cup [29,30]. Normal morphogenesis in this case is impeded if the ECM is softer than the cells. Moreover, the shape of the invagination is regulated by a stiffness gradient, since ECM is stiffer near the centre than at the margin [29,30].

In the case of the intestinal villi, a circular muscle layer forms a stiff constraint that physically prevents the free expansion of the mesenchyme and endoderm (Figure 1D) [31]. As these tissues grow, they compress, buckle and fold. In the absence of muscle differentiation, the gut tube expands freely and radially, indicating an absolute requirement of stiff constraints to correctly vilify the gut [31]. In flies, the wing imaginal disc (the precursor of the wing) forms folds as the tissue grows, because it buckles under high external resistance, which comes from a rigid apical domain and stiff basement membrane [32]. These rigid constraints contribute to enable precise and reproducible folding [32].

A similar story of tissues buckling as they grow within rigid borders is found during the morphogenesis of pavement cells within the outer epidermal layer of plants [33] and it has been proposed to contribute to the gyrification of the brain, with its characteristic wrinkling pattern [34].

Finally, it is important to highlight that because material properties and forces are intertwined, mechanical constraints can instruct cells how to direct force. For example, geometric constraints inherently contribute to specifying actomyosin configurations and orienting cell forces [35]. During *Drosophila* gastrulation, the rectangular shape (i.e. an anisotropic constraint) of the ventral furrow means more cells generate tension along the long (anteroposterior) axis than the short (dorsoventral) axis [35]. This orients the contractile actomyosin network and epithelial tension to specify proper tissue folding. By comparison, the more isotropically constrained domain of the presumptive posterior midgut results in cells having isotropic resistance, resulting in a pattern of force that is isotropic [35], leading to cup-like (rather than fold-like) invagination.

Altogether, these studies reveal that mechanical patterning of a developing tissue's surroundings directly and indirectly contributes to its final form, either by restricting or permitting deformation, or by contributing to correct force production for morphogenesis.

## Robustness of morphogenesis

Tissues develop very reproducibly in size and shape, a trait called robustness, that is characterised by low variation of a phenotype when subjected to environmental variations. Flexible and responsive tissue stiffness can help explain the robustness of development in the face of physical perturbations to which a tissue may be exposed.

**Figure 2.**
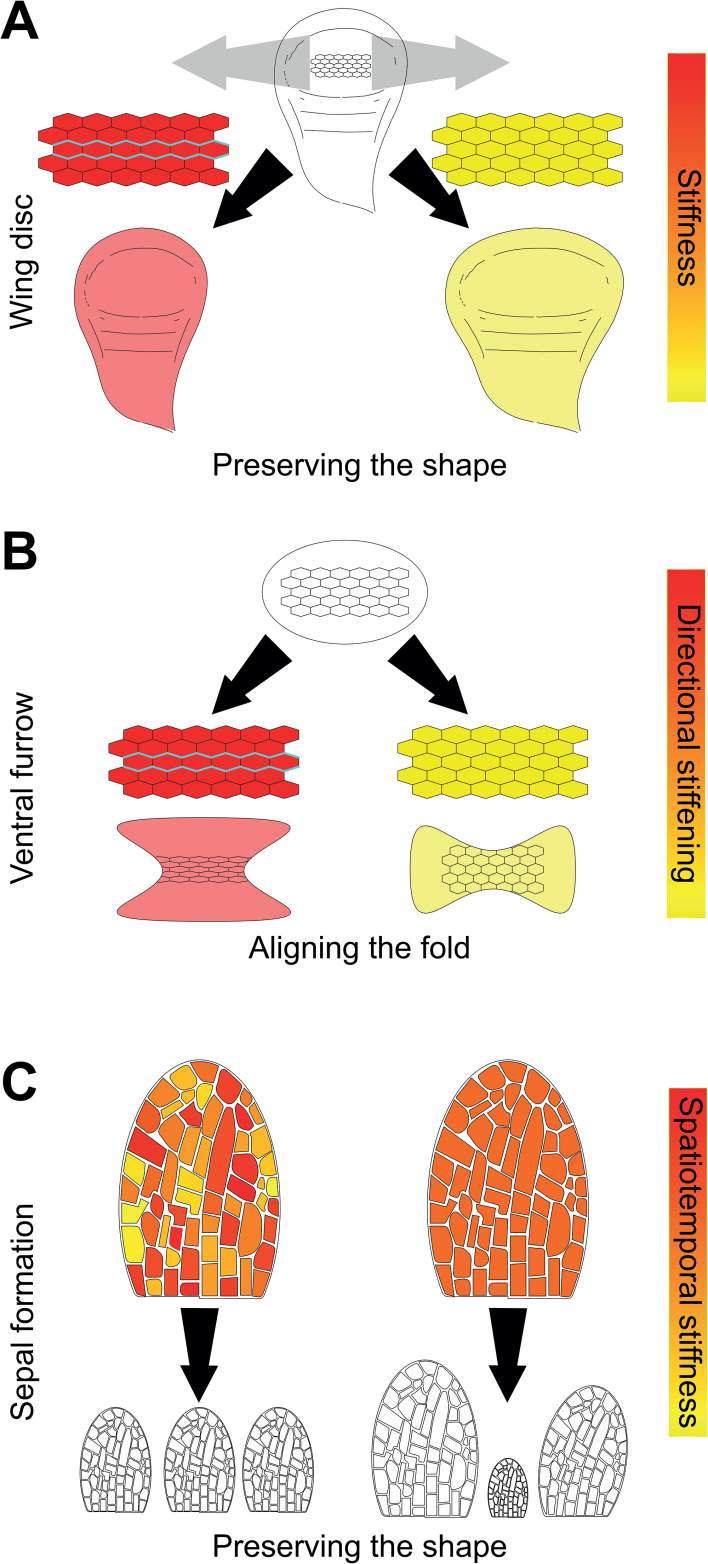
Variable stiffness enables robust morphogenesis. (**A**) If the fly wing disc (top) experiences a mechanical perturbation (grey arrows), the cells must dynamically increase their stiffness (red) via the formation of linear actomyosin cables (cyan) to preserve shape of the pouch (bottom left). If the cells do not stiffen, but rather remain compliant (yellow), the organ does not maintain its shape (right). (**B**) Cells in the ventral region of the *Drosophila* gastrula (top) invaginate via formation of a furrow. Normally, cells are connected via a supracellular actomyosin network and this directional rigidification enables the contractility to fold the tissue correctly (left). If the network does not have a directional bias, the contractility it exerts fails to correctly fold the tissue, instead producing a misaligned fold. (**C**) Sepal cells in the growing organ experience spatiotemporal variability in stiffness (left, top), allowing them to average their differences over time and space. The net result is an organ that grows reliably to its required shape and size (left, bottom). Uniformity of stiffness over space and time results in misshapen organs, because small errors are amplified and uncorrected.

As tissues stiffen, more force is required to change their form. Stiffer tissues thus limit shape changes and prevent fractures from propagating across them. In the fly wing, the actomyosin cytoskeleton reorganises into linear cables to increase tissue stiffness in response to extrinsic tensile forces (Figure 2A) [36]. This reaction allows the tissue to buffer forces and preserve its shape under mechanical stress [36]. During amphibian neurulation, axial tissues (primarily the neural plate) stiffen to overcome external resistance to elongation and maintain tissue integrity [37,38]. Cone cells in the retina also deform little when challenged with external mechanical perturbation, possibly through an increase in stiffness [39]. The ability of tissues to flexibly and dynamically change their rigidity thus ensures robust morphogenesis against environmental variation that might perturb growth and development.

As we discussed earlier, material properties and forces are intrinsically linked, and this has implications for morphogenetic robustness, such as in the well-studied model system of the ventral furrow. Mesodermal cells across the ventral portion of the tissue are connected by a supracellular cytoskeletal network. The connectivity of the network determines the tissue's rigidity. In a poorly connected network, the tissue is floppy and contractile forces are insufficient to enable folding, whereas a well-connected network can fold normally thanks to actomyosin contraction. Interestingly, stiffness (network connectivity) increases at the onset of invagination, beyond what would be minimally needed; allowing the system to be robust because if contractility was lower than normal, the morphogenetic process of tissue bending would be unimpeded [40]. Moreover, the network stiffens anisotropically: primarily along edges orthogonal to the folding axis (Figure 2B) [40]. This directional network stiffening changes the local bending energy of the tissue, meaning that is myosin-generated force can bend the tissue easier along the correct axis. Thus, anisotropic stiffness also enables robust furrow formation by preventing misoriented folding. Hence, spatiotemporal stiffening ensures reliable tissue folding (by myosin contractility) with proper orientation, highlighting that mechanical redundancy allows tissues to undergo morphogenesis despite potential damage. Stiffening of the actomyosin network along edges orthogonal to the folding axis isolates the fold domain from surrounding mechanical perturbations, preventing force scattering when myosin contracts [41].

Finally, another concept that may widely explain the ability of developing tissues to overcome noise was discovered in a study of plant morphogenesis. Sepal epidermal cells show significant variability in their stiffness, both in space and in time [42]. Spatiotemporal variability allows differences in stiffness to be averaged over time so, overall, the sepal grows with uniform stiffness. In the absence of temporal variability, organs are misshapen, because small errors are multiplied by all the cells homogeneously having the same error, resulting in an exaggerated size and shape [42]. Therefore, counterintuitively, more uniformity in cell growth between neighbours of the developing sepal disrupts the final size and shape of the organ; whereas high variability in stiffness across the tissue promotes sepal shape robustness. Spatiotemporal averaging of rigidity to overcome noise in morphogenetic systems may be a common biological process, analogous to how molecular processes, like stochastic transcriptional activity, have been proposed [43].

## Mechanical patterns for self-organisation

Many aspects of morphogenesis emerge from self-organisation. The oldest and most established explanation of self-organisation is Turing's reaction-diffusion system, in which patterns arise from diffusion of activator and inhibitor, namely, long-range inhibition and short-range activation [44]. Such a system was successful in identifying chemical patterns of self-organisation. Recently, there has been evidence of physical cues acting to self-organise morphogenesis in a similar way.

Theoretical work suggests that if a cell layer is highly contractile, it can cross an instability threshold, whereby the forces are so large that it ‘pulls itself apart’ [45,46]. This leads to a heterogeneous configuration of cells. Therefore, the amount of contraction and, significantly, the amount of resistance to contraction — provided by the stiffness of the substrate — affect the spacing of cellular aggregates. Larger contraction gives rise to larger spacing, whereas more resistance leads to shorter spacing. This means that a regular pattern of cellular aggregates can emerge from an initially uniform population of cells; a process which depends on the mechanical properties of the cells themselves and the rigidity of the surrounding extracellular environment.

**Figure 3.**
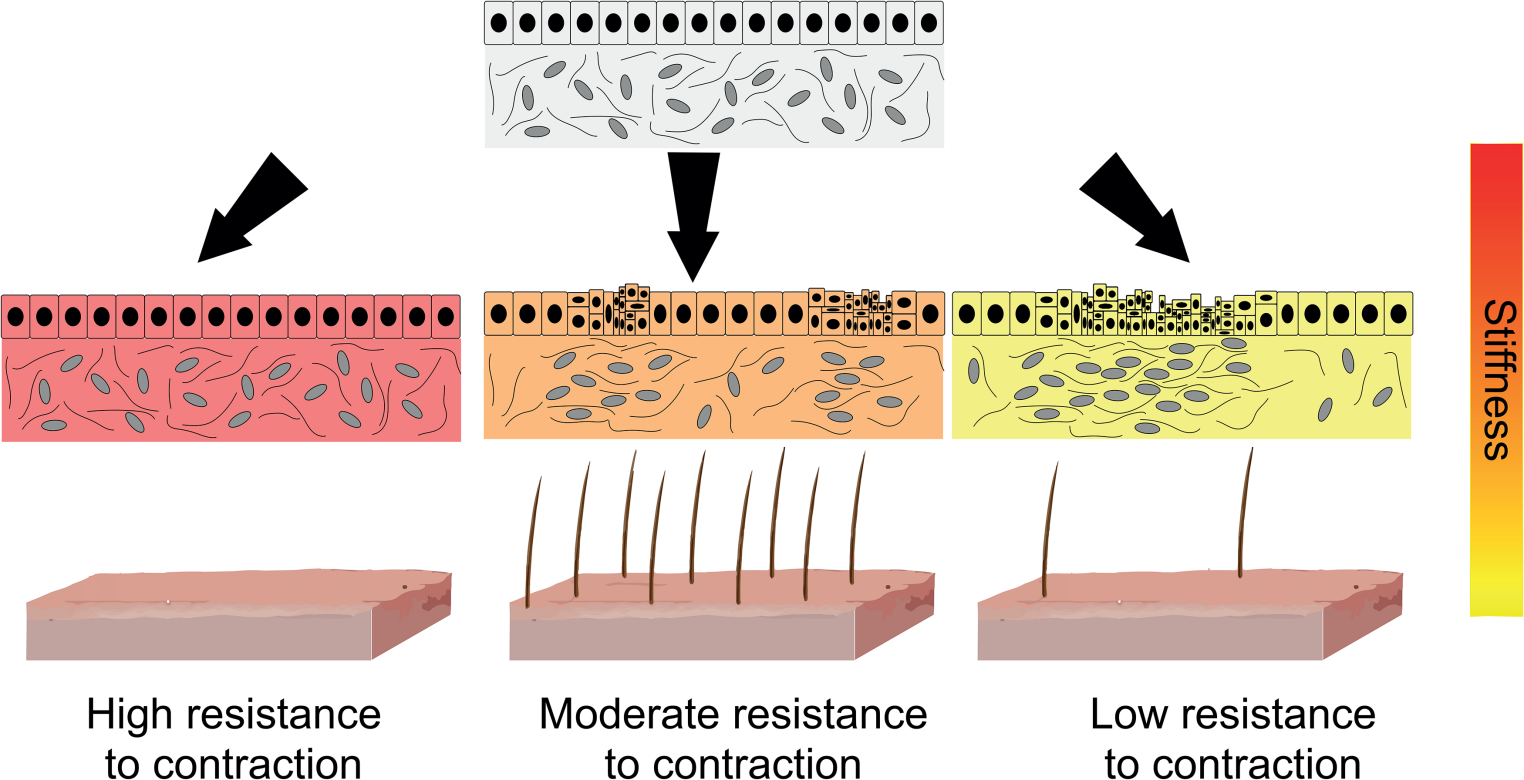
Mechanical resistance as a long-range inhibitor in patterning. The skin comprises of an epidermal cell layer on top of the dermis, which comprises contractile cells and extracellular matrix (top). The dermis undergoes contractile instability to form aggregates when the stiffness is not too resistive (middle), which compresses the overlying epidermis, which will form placodes and hair or feathers in a patterned manner. However, if it is too rigid (red), the contractile forces cannot overcome the stiffness and aggregates will not form (left). On the other hand, if it is too soft (yellow), it will collapse into a single aggregate as contractility overpowers resistance.

Theoretical models of this idea were hypothesised decades ago. George Oster hypothesised that changes in cell motility and stiffness could give rise to different self-emergent patterns from a uniform field of cells [45]. Experimental evidence for mechanical patterns has been provided recently in the context of placode formation in the developing skin. The dermis undergoes contractile instability and forms aggregates, which result in compression of the overlying epidermis, where mechanotransduction initiates feather bud gene expression in chick embryos (Figure 3) [47]. Substrate stiffness is therefore a critical regulator of self-organised morphogenesis; in this case, it serves as a long-range inhibitor of follicle pattern formation [47]. There is also evidence for stiffness-dependent morphogenesis of hair placodes across the skin in mice [48]. Although absolute values of stiffness vary between mouse species, a mechanical reaction-diffusion setup elegantly explains differences in the regeneration of hair follicles in different species and is predicated on the setup of the morphogenetic field and placode formation, matching the Turing model that has been proposed to underly pattern formation [48]. Indeed, perturbing tissue stiffness is sufficient to change the shape of the competent field.

These recent findings reveal how mechanical feedback in a Turing-style reaction-diffusion model enables self-organisation, implying that stiffness, and potentially other mechanical cues, help orchestrate morphogenesis in a robust, reproducible manner and enable efficient patterning.

## Migration and invasion

**Figure 4.**
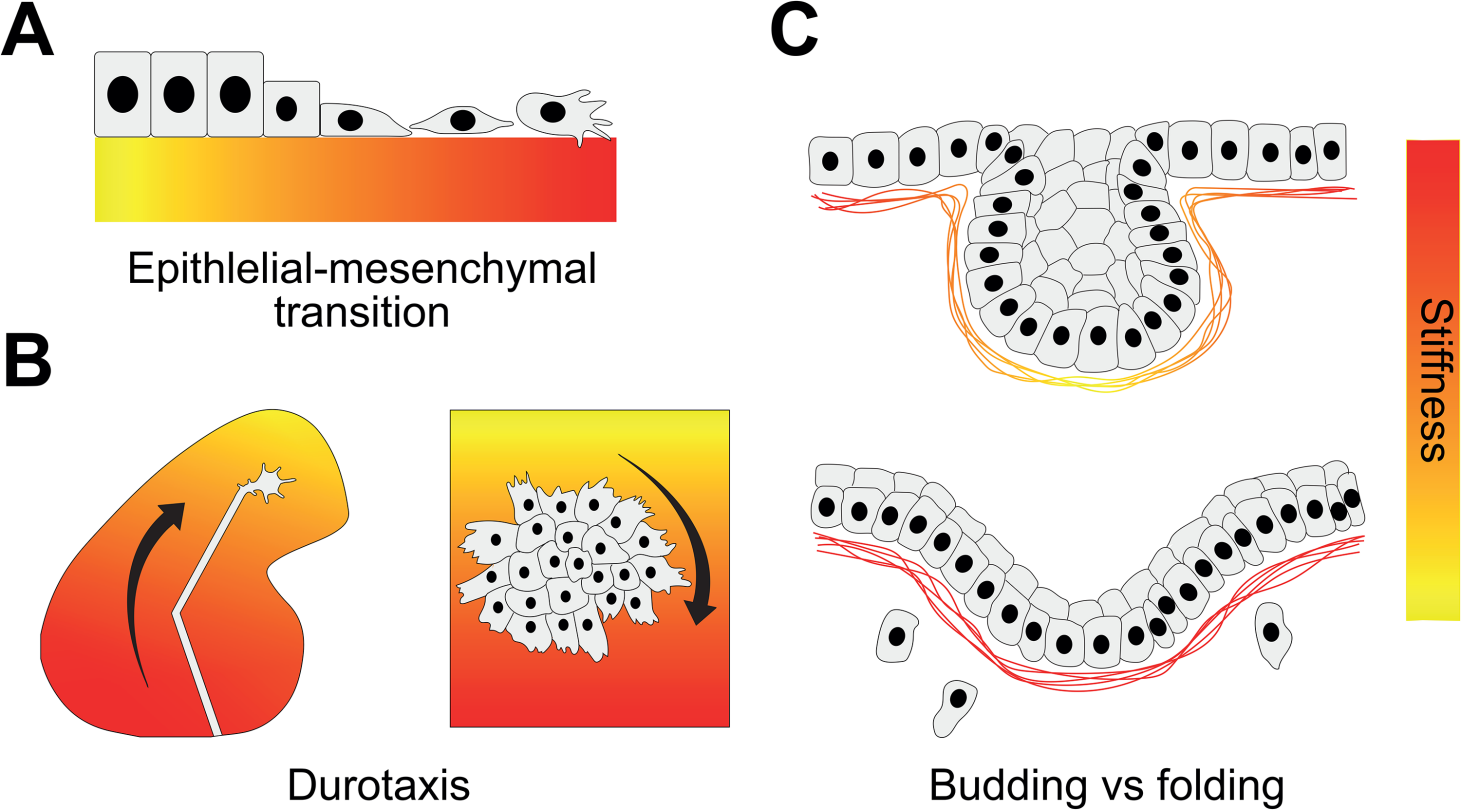
Substrate rigidity co-ordinates migration and tumorigenesis. (**A**) Soft substrates promote an epithelial phenotype, cells mechanosense stiff substrates, reprogramming as part of an epithelial-to-mesenchymal transition to become motile and invasive. (**B**) Cells can feel the stiffness of their substrate and are guided by stiffness gradients. Axons in the developing eye grow toward soft (yellow) substrate (left), whereas neural crest cells collectively move toward stiff (red) substrate (right). (**C**) Benign epidermal cells degrade the basement membrane, which makes it soft, allowing the formation of a bud (top). Malignant epidermal cells remodel the basement membrane less, resulting in a fold-like shaped tumour that is unable to overcome the resistive force of the stiff basement membrane (bottom). The authors suggest these types of tumours may eventually rupture to enable invasion as single cells.

During animal development, cells are often not specified where they are later destined to be. Hence, a key aspect of morphogenesis involves the reorganisation of tissues and migration of cells from one location to another. The potential for cells to begin migrating is usually preceded by an epithelial-to-mesenchymal transition (EMT), the process by which epithelial cells lose their apicobasal polarity in favour of front-rear polarity, weaken their intercellular adhesions and become motile. Cellular EMT is impacted by cell's mechanosensing changes in the rigidity of their surroundings, with an increase in stiffness triggering EMT (Figure 4A) [49,50]. This has implications in tumorigenesis and embryonic development, since EMT in both cancer cells and neural crest cells is impacted in this manner.

Once motile, cells physically pull on their surroundings. In doing so, they detect the amount of resistance that is being provided to the pulling force, thereby allowing them to sense the stiffness of nearby fibres and cells. Because stiff materials deform less than soft materials, cells are typically naturally guided to follow stiffness gradients toward stiffer substrate, a process called durotaxis [51]. An analogy to explain the physical mechanism of this behaviour is that of a person on a skateboard that is equally pulling on a stiff spring in one hand and a flexible spring in the other. The stiff spring will deform and the flexible spring will not, meaning the skateboard will roll toward the stiff spring [52]. Durotaxis occurs in embryogenesis, enabling neural crest cells to reach their destination by following the stiffness gradient of their substrate, which is the adjacent placodal tissue (Figure 4B, left) [53]. Stiffness gradients also control the directionality of axon growth in the developing brain (Figure 4B, right) [54] and may contribute to limb bud morphogenesis [55] and tumorigenesis [56].

Tissue invasion is also mediated by the presence of absence of perforations in the basement membrane. For example, reductions in stiffness permit the formation of salivary gland epithelial end buds and subsequent branching morphogenesis [57]. In tumorigenesis, cancer-associated fibroblasts (CAFs) guide cancer cells out of the primary tumour site to secondary locations by pulling, stretching and softening the basement membrane, leading to the formation of gaps through which cancer cells can migrate [58]. CAFs thus alter the physical properties of the basement membrane, making it permissive for cancer cell invasion [58].

Basement membrane stiffness also controls the cellular architecture and growth of cancer progression. When the basement membrane is soft, and as benign carcinomas become overcrowded as they grow and proliferate, the basement membrane buckles, creating a bud-shaped, benign tumour (Figure 4C, top) [59]. On the other hand, if the tumour grows beside a rigid basement membrane, it is unable to dissipate the compressive forces it exerts, producing wave-like folds (Figure 4C, bottom) [59]. Thus, tumour morphogenesis can be directed by extracellular rigidity.

## Morphogenetic flows

During embryogenesis, tissue layers undergo dramatic changes that change their topology and shape. These processes involve a coherent flow of cells and cell rearrangements, a process generally termed morphogenetic flows. Morphogenetic flows contribute to rearranging and folding of tissues into specific shapes, and regional spatially varying control of tissue rigidity is a key physical mechanism that guides morphogenetic flows.

**Figure 5.**
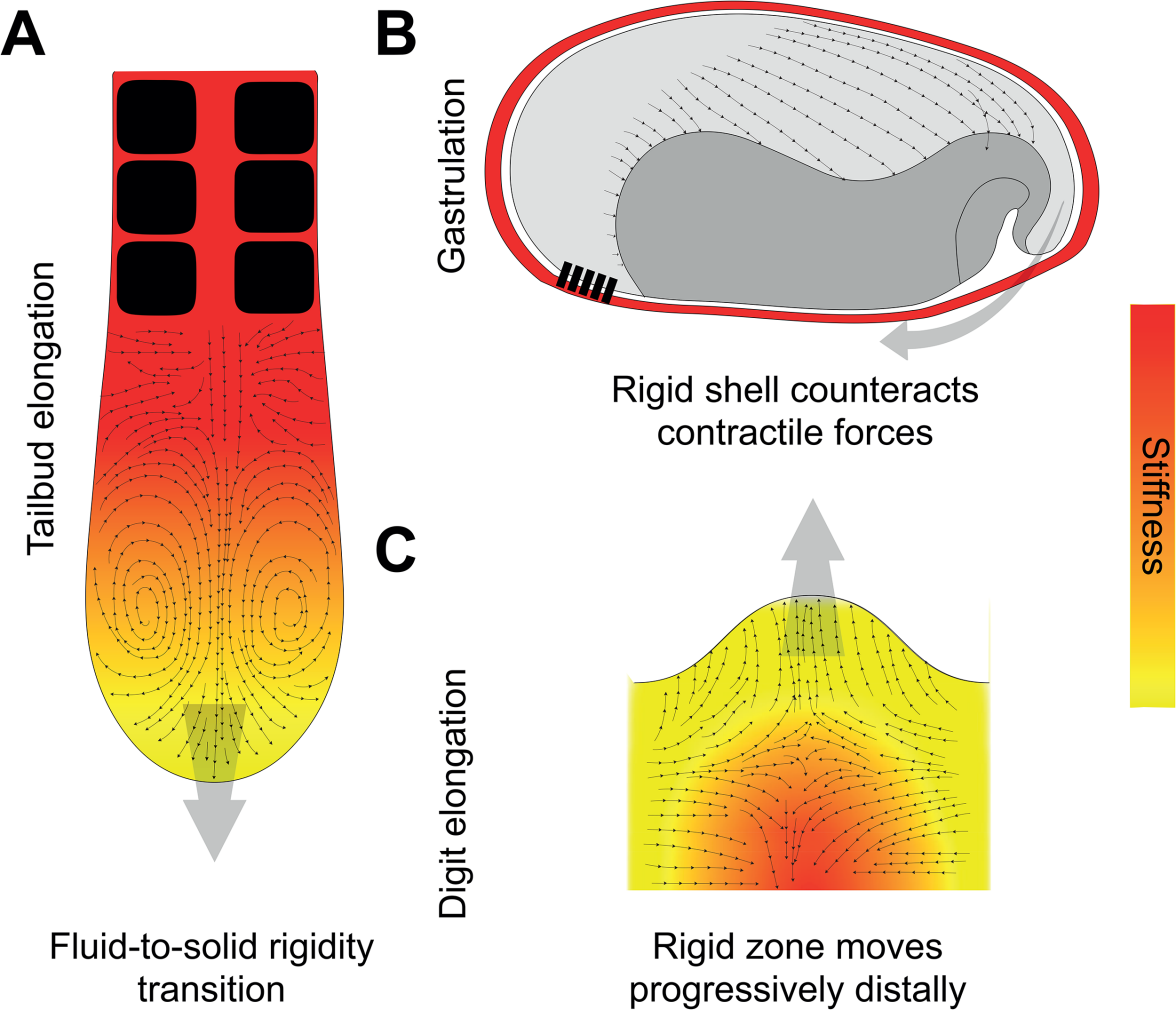
Rigidity transitions co-ordinate morphogenetic flows. (**A**) The vertebrate body axis has a rigidity gradient, in which cells near the tailbud are soft (yellow) and fluid, and those further back are rigid (red). The fluid-to-solid tissue transition can explain the observed morphogenetic flows (black arrows) consisting of two counter-rotating vortices, that enable axis elongation (grey arrow). (**B**) Gastrulation in beetles is characterised by large-scale posterior flow of the dorsal tissue. This flow is thanks to asymmetric attachment of the blastoderm to the overlying vitelline membrane, which act as a rigid shell. Attachment (black rectangles) of the ventral side to the rigid shell enables it to counter-act intrinsic contractile forces, whereas the dorsal tissue is unattached and thus flows freely. (**C**) Digit elongation is characterised by a morphogenetic flow that emerges from a rigid zone that moves distally over time, similar to axis elongation.

In the case of vertebrate axis elongation, anterior tissues are stiff, mechanically supporting the extending body axis, whereas posterior tissues are fluid, exhibiting zero stiffness, which enables the tissue to locally remodel in the elongating tailbud region, with cells able to move freely and deform easily (Figure 5A) [60]. The rigidity transition is explained by the geometry and density of the cells within the tissue, where cells far away from the tailbud are packed [60]. This affects local tissue stiffness, controlling the solidification process, and is essential to account for the flow fields, as the tissue transits from fluid to solid states [11]. Similarly, changes in network rigidity underpin the development of the zebrafish blastoderm, a tissue which spreads over the yolk at the onset of gastrulation, and which is hallmarked by a rigidity transition caused by a reduction in the cellular connectivity, turning a rigid network with high intercellular adhesions in a floppy one with low intercellular adhesions [61].

Extensive morphogenetic movements are also seen during gastrulation. During the initial phases of gastrulation in *Tribolium* beetles the morphogenetic flows are characterised by a large-scale posterior flow of dorsal tissue, with blastoderm sliding freely underneath the vitelline membrane, whereas anteroventral tissue (on the opposite side of the embryo) remains stationary [62,63]. These features collectively result in large-scale unidirectional flow. These flows arise thanks to anchoring of anteroventral blastoderm on the vitelline envelope, which acts as a rigid shell that counteracts tissue-intrinsic contractile forces to generate asymmetric flows during gastrulation (Figure 5B) [64]. Modelling the anchor points of the blastoderm to the vitelline membrane with infinite friction matches experimental observations of the flow fields [64]. Anchorage to rigid shells can drive the morphogenetic flows of many other events including endoderm morphogenesis [65] and formation of the correct wing shape [66]. In the latter's case, the pupal wing attaches to the rigid ECM distally, enabling it to create an anisotropic force along the wing blade that extends and shapes the organ. Thus, the pattern of stiff extracellular components, and correct anchorage of cells to it to counteract tissue-intrinsic forces, is fundamental in determining the correct axis for tissue elongation and normal morphogenesis [66,67].

These general ideas are now being extended to more classical systems that have previously been explained by molecular self-organisation processes, such as digit formation. Convergent extension tissue flows underlying digit formation are driven by a compressed region that progressively moves further distal due to local rigidification of tissue in digit-organising centres (Figure 5C) [68], much like vertebrate axis elongation.

## Feeding back: forces affecting material properties

We introduced this review by explaining how morphogenesis is a consequence of material properties and forces, and we then described several ideas related to how stiffens can direct morphogenesis. With reference to examples we mentioned earlier, we would now like to briefly address feedback in the opposite way: how the forces of morphogenesis affect material properties. Although stiffness is, in many cases, an intrinsic property of tissues, there is growing evidence of how forces can modulate stiffness. We explored some of these ideas earlier, in the context of external stress, during which tissues can respond by changing their material properties, thereby buffering the forces and preserving tissue integrity (Figure 2).

Mechanical forces regulating stiffness is not only limited to external physical perturbations, but also happens during normal morphogenesis too. Earlier, we mentioned that neural crest cells follow a gradient of stiffness (a process called durotaxis) in the developing embryo. This stiffness gradient emerges because the neural crest cells themselves soften the adjacent placodal tissue via forces from cell–cell adhesions [53], creating soft surroundings nearby and stiffer surroundings ahead. Border cells — which also collectively migrate — likewise modulate substrate stiffness, in this case stiffening the adjacent nurse cells [69]. In both cases, failure to modulate substrate stiffness impairs collective migration, and there is thus a cyclical feedback between tissue morphogenesis and stiffness.

Forces can also affect stiffness of extracellular material, although, it should be noted that mechanical properties of ECM in general are complex, and the ECM is viscoelastic, meaning it deforms over time under an applied stress and can return to its original shape when the stress is removed. Thus, stiffness alone is only one metric amongst many that should be considered. For example, if degradation occurs slowly and in a controlled manner, as it does during processes like tissue repair, mechanical changes may be subtle or even negligible.

We also highlight the model system of placodal patterning, which arises from mechanical self-organisation. Stiffness in the dermis (an essential modulator of placodal morphogenesis) arises from reciprocal cell-ECM interactions: cells pull on fibres, which makes them more contractile, leading to higher rigidity and contractile instability [70]. We also earlier described how basement membrane stiffness can influence the mode of cancer morphogenesis, promoting buds or folds. Equally, though, tumour morphogenesis guides basement membrane rigidity. In benign carcinomas, epidermal cells remodel the basement membrane, which can make it soft, whereas malignant tumours remodel the basement membrane less, which can cause it to be comparatively rigid [42]. In this case, the wave-like folds the tumour will eventually form may rupture the basement membrane, enabling invasion.

Collectively, these examples highlight the complexity of the mechanics of morphogenesis.

## Outlook

Morphogenesis across species gives rise to an enormous array of tissues and organs. We hope that this mini-review has highlighted the critical importance that material properties, like stiffness, has on orchestrating morphogenesis and the formation of diverse tissues.

We anticipate further research on the role of material properties will provide a cohesive physical explanation underpinning morphogenesis. Morphogenetic events do not happen in isolation, but require large-scale co-ordination so understanding the rigidity changes locally is insufficient to explain such processes. One notable example in which there has been considerable progress to gain a coherent view is in the ventral furrow. We have discussed many of the local behaviours at play, but there is also an appreciation that other parts of the embryonic epithelium must respond or contribute to the movement of ventral invagination in a concerted manner. Tissues on the lateral sides of the embryo need to move toward the furrow and the tissue on the (opposite) dorsal side needs to stretch and expand, made feasible by softening, which is molecularly explained by a reduction in actomyosin levels [71,72]. This enables the mesoderm to fold and invaginate on the ventral side. In contrast, lateral tissues remain stiff, with a dense medial-apical actomyosin network.

Given the impressive amount we have learnt from studying stiffness, there are plenty of other material properties that can garner attention in the next few years. These include viscosity (a strain rate dependent on time) stress-relaxation (the observed time-dependent reduction in stress in response to constant strain), creep (a time-dependent strain at constant stress), strain-rate sensitivity (change in strain over time) and hysteresis (a difference in the stress-strain relation when loading/unloading due to viscoelasticity). Assaying material properties in developing organisms is very difficult, though, and progress will be catalysed by the rate of development of new technologies.

Further progress in this field will be accelerated by methods that enable rapid, non-invasive, non-destructive mechanical imaging. Considerable progress has been made with techniques like Brillouin microscopy, which offers the ability to study the viscoelastic properties of biological samples in 3D over space and time. Brillouin microscopy has recently been impressively applied to various model systems in the context of morphogenetic events like tissue folding [73]. Although further research is needed to fully characterise the mechanical properties being measured by such methods [74], technologies like this provide exciting new possibilities to better characterise material properties of developing biological systems.

## Perspectives

- Material properties of tissues have been increasingly well recognised as an important component for understanding how form of tissues emerges. In many cases, this has resolved discrepancies that have existed purely based on gene expression patterns.
- Stiffness of tissues plays an active role in morphogenetic form. It can constrain or permit growth and migration, dictate morphogenetic flows, enable Turing-like patterning and contribute to robustness.
- The development of new technologies will spur research into other material properties in attempt to gain a holistic understanding of the physical basis of morphogenesis.

## Open Access

Open access for this article was enabled by the participation of University College London in an all-inclusive *Read & Publish* agreement with Portland Press and the Biochemical Society under a transformative agreement with JISC.

## Abbreviation

CAFs: cancer-associated fibroblasts
EMT: epithelial-to-mesenchymal transition
